# 6-Hy­droxy-1,2-dihydro-4*H*-pyrrolo­[3,2,1-*ij*]quinolin-4-one

**DOI:** 10.1107/S1600536813003000

**Published:** 2013-02-02

**Authors:** Victor B. Rybakov, Svitlana V. Shishkina, Igor V. Ukrainets, Olga V. Gorokhova, Xeniya V. Andreeva

**Affiliations:** a Department of Chemistry, Moscow State University, Moscow 119992, Russian Federation; b STC "Institute for Single Crystals", National Academy of Sciences of Ukraine, 60 Lenina ave., Kharkiv 61001, Ukraine; c National University of Pharmacy, 4 Blyukhera St., Kharkiv 61002, Ukraine

## Abstract

The mol­ecule of the title compound, C_11_H_9_NO_2_, is essentially planar [r.m.s. deviation of the non-H atoms = 0.056 (1) Å]. In the crystal, strong O—H⋯O hydrogen bonds form zigzag chains along the *b* axis. The mol­ecules form stacks along the *a* axis due to π–π inter­actions, the shortest distance between the centroids of the benzene and pyridinone rings being 3.6146 (7) Å.

## Related literature

For condensation of secondary anilines with triethyl methane­tricarboxyl­ate, see: Kutyrev & Kappe (1997 ▶); Jönsson *et al.* (2004 ▶); Ukrainets *et al.* (2006 ▶, 2010 ▶, 2011 ▶). For standard bond lengths, see: Allen *et al.* (1987 ▶). For a related structure, see: Baumer *et al.* (2004 ▶).
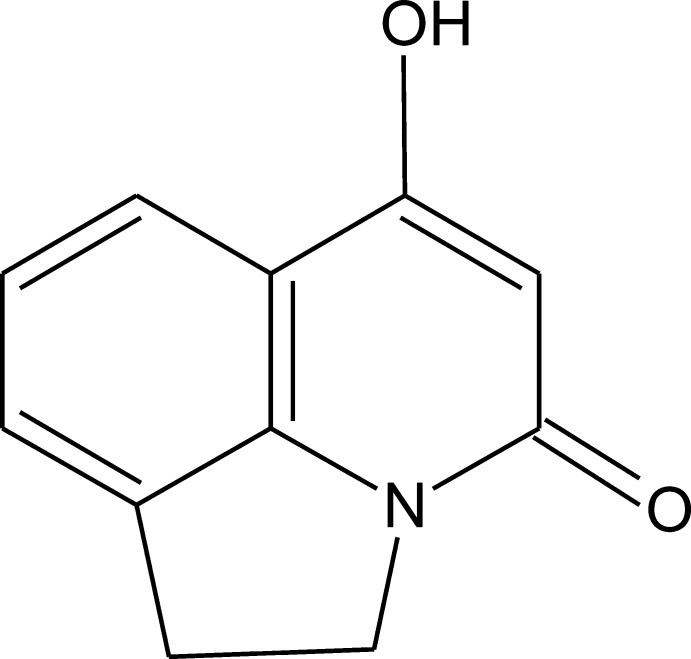


## Experimental

### 

#### Crystal data

C_11_H_9_NO_2_
*M* *_r_* = 187.19
Monoclinic, 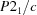

*a* = 7.9987 (3) Å
*b* = 7.6297 (2) Å
*c* = 14.3500 (4) Å
β = 101.386 (3)°
*V* = 858.51 (5) Å^3^
*Z* = 4
Mo *K*α radiation
μ = 0.10 mm^−1^
*T* = 295 K
0.20 × 0.10 × 0.10 mm

#### Data collection

Agilent Xcalibur Sapphire3 CCD diffractometer
Absorption correction: multi-scan (*CrysAlis RED*; Agilent, 2011 ▶) *T*_min_ = 0.983, *T*_max_ = 1.000
7610 measured reflections
2501 independent reflections
1806 reflections with *I* > 2σ(*I*)
*R*_int_ = 0.023

#### Refinement

*R*[*F* ^2^ > 2σ(*F* ^2^)] = 0.044
*wR*(*F* ^2^) = 0.125
*S* = 1.07
2501 reflections
131 parameters
H atoms treated by a mixture of independent and constrained refinement
Δρ_max_ = 0.23 e Å^−3^
Δρ_min_ = −0.15 e Å^−3^

### 

Data collection: *CrysAlis CCD* (Agilent, 2011 ▶); cell refinement: *CrysAlis CCD*; data reduction: *CrysAlis RED* (Agilent, 2011 ▶); program(s) used to solve structure: *SHELXS97* (Sheldrick, 2008 ▶); program(s) used to refine structure: *SHELXL97* (Sheldrick, 2008 ▶); molecular graphics: *ORTEP-3 for Windows* (Farrugia, 2012 ▶); software used to prepare material for publication: *WinGX* (Farrugia, 2012 ▶).

## Supplementary Material

Crystal structure: contains datablock(s) I, global. DOI: 10.1107/S1600536813003000/yk2087sup1.cif

Structure factors: contains datablock(s) I. DOI: 10.1107/S1600536813003000/yk2087Isup2.hkl

Additional supplementary materials:  crystallographic information; 3D view; checkCIF report

## Figures and Tables

**Table 1:** Hydrogen-bond geometry (Å, °)

*D*—H⋯*A*	*D*—H	H⋯*A*	*D*⋯*A*	*D*—H⋯*A*
O2—H2⋯O1^i^	1.09 (2)	1.51 (2)	2.5922 (13)	172 (2)

Symmetry code: (i) 
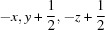
.

**Fig. 1.**
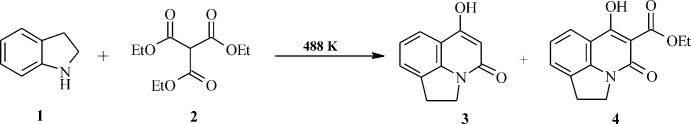
Synthesis path to the title compound.

**Fig. 2.**
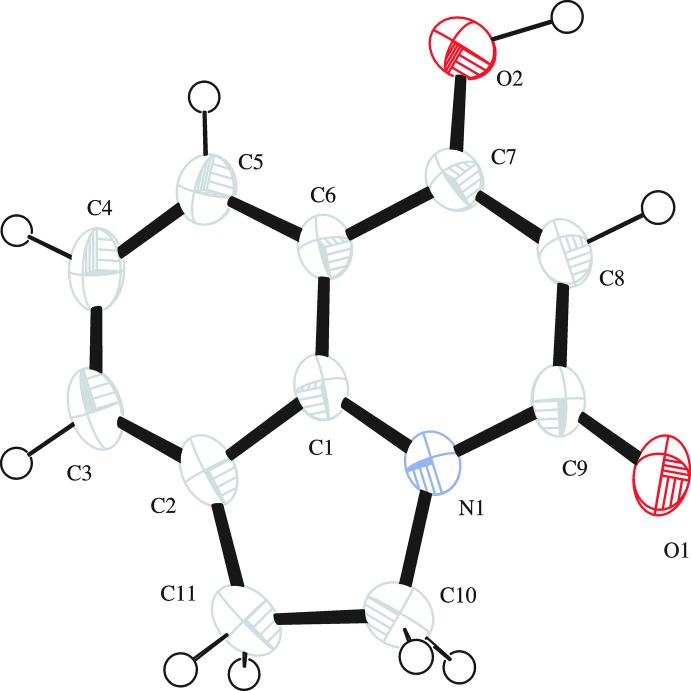
View of the title compound with the atom numbering scheme. The displacement ellipsoids are drawn at the 50% probability level. H atoms are presented as a small spheres of arbitrary radius.

**Fig. 3.**
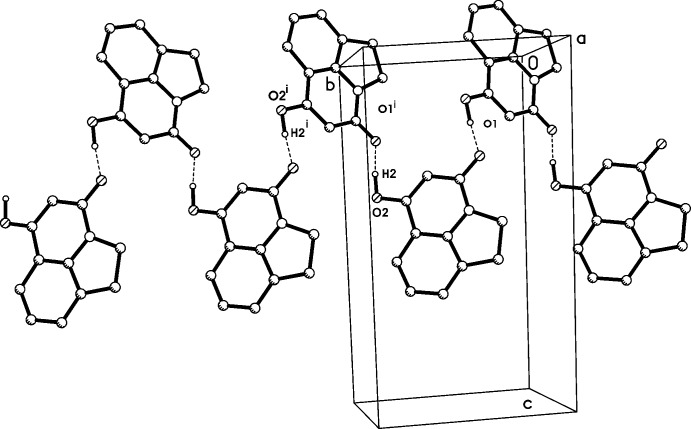
The O—H···O hydrogen bonds in the crystals of the title compound (shown by dashed lines). Symmetry code: (i) -x, y+1/2, -z+1/2.

## supplementary crystallographic information

### Comment

By now the most convenient method of obtaining ethyl esters of *N*-substituted 4-hydroxy-2-oxo-1,2-dihydroquinoline-3-carboxylic acids which are widely used in synthesis of the biologically active substances is condensation of the corresponding secondary anilines with triethyl methanetricarboxylate (Kutyrev & Kappe, 1997; Jönsson *et al.*, 2004; Ukrainets *et al.*, 2006; 2010; 2011). The method is efficient and gives higher yields. However, as it turned out, in such reactions specific by-products of the same type are also formed besides the targeted esters - (usually 2%–6% by *HPLC*). Taking condensation of indoline (1) with triethyl methanetricarboxylate (2) as an example (Fig. 1), we showed that in this case the by-product is 6-hydroxy-1,2-dihydro-4*H*-pyrrolo[3,2,1-*i,j*]quinolin-4-one (3), and its yield is determined by water content in the initial reaction mixture. The source of this impurity can be our main product - ethyl 6-hydroxy-4-oxo-1,2-dihydro-4*H*-pyrrolo[3,2,1-*i,j*]- quinoline-5-carboxylate (4), which readily undergoes partial hydrolysis and then decarboxylation at high temperature.

In the title molecule, C_11_H_9_NO_2_, the heterotricycle is essentially planar (Fig. 2). The bond lengths and angles are within the normal ranges (Allen *et al.*, 1987). Strong O2—H2···O1^i^ intermolecular hydrogen bonds (Table 1) form folded chains along the *b* axis (Fig. 3). Symmetry code: (i) -*x*, *y* + 1/2, -*z* + 1/2.

### Experimental

As shown in Fig. 1, Indoline (1) (11.2 ml, 0.1 mol) was added dropwise with stirring to triethyl methanetricarboxylate (2) (63.3 ml, 0.3 mol) heated to 488 K, at such a rate that the temperature of the reaction mixture was maintained within ±5 K of the initial temperature. The ethanol eliminated during the reaction was distilled through a suitable still–head. After adding all the indoline, the reaction mixture was kept at the same temperature for 30 min, after which it was cooled. The excess of triethyl methanetricarboxylate was removed in *vacuo*. To the residue was added 50 ml of xylene. The insoluble solid quinolin-4-one (3) was filtered off, washed with hexane, and dried. Yield: 0.77 g (4.1%). *M*.p. 577–579 K (*DMF*).

### Refinement

The H atom of hydroxyl group was located from electron density difference map and refined isotropically. The methylene and aromatic H atoms were placed in calculated positions and refined in the riding model approximation with C—H = 0.97 Å for methylene and C—H = 0.93 Å for aryl H atoms with *U*_iso_(H) = 1.2*U*_eq_(C).

### Crystal data

**Table:**

C_11_H_9_NO_2_	*F*(000) = 392
*M**_r_* = 187.19	*D*_x_ = 1.448 Mg m^−^^3^
Monoclinic, *P*2_1_/*c*	Melting point = 577–579 K
Hall symbol: -P 2ybc	Mo *K*α radiation, λ = 0.71073 Å
*a* = 7.9987 (3) Å	Cell parameters from 2056 reflections
*b* = 7.6297 (2) Å	θ = 3.0–30.0°
*c* = 14.3500 (4) Å	µ = 0.10 mm^−^^1^
β = 101.386 (3)°	*T* = 295 K
*V* = 858.51 (5) Å^3^	Prism, colourless
*Z* = 4	0.20 × 0.10 × 0.10 mm

### Data collection

**Table:**

Agilent Xcalibur Sapphire3 CCD diffractometer	2501 independent reflections
Radiation source: Enhance (Mo) X–ray Source	1806 reflections with *I* > 2σ(*I*)
Graphite monochromator	*R*_int_ = 0.023
Detector resolution: 16.1827 pixels mm^-1^	θ_max_ = 30.0°, θ_min_ = 3.0°
ω scans	*h* = −10→11
Absorption correction: multi-scan (*CrysAlis RED*; Agilent, 2011)	*k* = −10→10
*T*_min_ = 0.983, *T*_max_ = 1.000	*l* = −20→19
7610 measured reflections	

### Refinement

**Table:**

Refinement on *F*^2^	Primary atom site location: structure-invariant direct methods
Least-squares matrix: full	Secondary atom site location: difference Fourier map
*R*[*F*^2^ > 2σ(*F*^2^)] = 0.044	Hydrogen site location: inferred from neighbouring sites
*wR*(*F*^2^) = 0.125	H atoms treated by a mixture of independent and constrained refinement
*S* = 1.07	*w* = 1/[σ^2^(*F*_o_^2^) + (0.0595*P*)^2^ + 0.0832*P*] where *P* = (*F*_o_^2^ + 2*F*_c_^2^)/3
2501 reflections	(Δ/σ)_max_ < 0.001
131 parameters	Δρ_max_ = 0.23 e Å^−^^3^
0 restraints	Δρ_min_ = −0.15 e Å^−^^3^

### Special details

**Table:**

Experimental. *CrysAlis RED* (Agilent Technologies, 2011). Empirical absorption correction using spherical harmonics, implemented in *SCALE3 ABSPACK* scaling algorithm.
Geometry. All s.u.'s (except the s.u. in the dihedral angle between two l.s. planes) are estimated using the full covariance matrix. The cell s.u.'s are taken into account individually in the estimation of s.u.'s in distances, angles and torsion angles; correlations between s.u.'s in cell parameters are only used when they are defined by crystal symmetry. An approximate (isotropic) treatment of cell s.u.'s is used for estimating s.u.'s involving l.s. planes.
Refinement. Refinement of *F*^2^ against ALL reflections. The weighted *R*–factor w*R*and goodness of fit *S* are based on *F*^2^, conventional *R*–factors *R* are based on *F*, with *F* set to zero for negative *F*^2^. The threshold expression of *F*^2^ > 2σ(*F*^2^) is used only for calculating *R*–factors(gt) *etc*. and is not relevant to the choice of reflections for refinement. *R*–factors based on *F*^2^ are statistically about twice as large as those based on *F*, and *R*–factors based on ALL data will be even larger.

### Fractional atomic coordinates and isotropic or equivalent isotropic displacement parameters (Å^2^)

**Table:**

	*x*	*y*	*z*	*U*_iso_*/*U*_eq_	
O1	0.20058 (12)	0.29009 (13)	0.28449 (6)	0.0523 (3)	
O2	−0.01907 (13)	0.82083 (12)	0.38558 (7)	0.0489 (3)	
H2	−0.093 (3)	0.819 (3)	0.3132 (16)	0.101 (7)*	
N1	0.26368 (13)	0.36896 (13)	0.43936 (7)	0.0369 (2)	
C1	0.25940 (14)	0.48889 (16)	0.51022 (8)	0.0345 (3)	
C2	0.35421 (15)	0.43260 (18)	0.59709 (8)	0.0407 (3)	
C3	0.36190 (17)	0.5414 (2)	0.67396 (9)	0.0507 (4)	
H3	0.4236	0.5086	0.7333	0.061*	
C4	0.27589 (18)	0.7023 (2)	0.66225 (10)	0.0519 (4)	
H4	0.2836	0.7763	0.7144	0.062*	
C5	0.18033 (17)	0.75496 (18)	0.57616 (9)	0.0439 (3)	
H5	0.1230	0.8617	0.5709	0.053*	
C6	0.17035 (15)	0.64553 (16)	0.49625 (8)	0.0354 (3)	
C7	0.07582 (15)	0.67526 (15)	0.40090 (8)	0.0362 (3)	
C8	0.08626 (15)	0.55614 (16)	0.33158 (8)	0.0391 (3)	
H8	0.0262	0.5785	0.2704	0.047*	
C9	0.18435 (15)	0.39879 (16)	0.34778 (8)	0.0379 (3)	
C10	0.36451 (18)	0.21387 (18)	0.47642 (10)	0.0479 (3)	
H10B	0.4609	0.1991	0.4454	0.057*	
H10A	0.2951	0.1087	0.4670	0.057*	
C11	0.42503 (18)	0.25294 (19)	0.58354 (10)	0.0487 (3)	
H11B	0.3808	0.1665	0.6219	0.058*	
H11A	0.5486	0.2530	0.6007	0.058*	

### Atomic displacement parameters (Å^2^)

**Table:**

	*U*^11^	*U*^22^	*U*^33^	*U*^12^	*U*^13^	*U*^23^
O1	0.0555 (6)	0.0572 (6)	0.0394 (5)	0.0028 (5)	−0.0024 (4)	−0.0165 (4)
O2	0.0565 (6)	0.0446 (5)	0.0426 (5)	0.0102 (4)	0.0021 (4)	0.0046 (4)
N1	0.0369 (5)	0.0396 (5)	0.0321 (5)	0.0004 (4)	0.0017 (4)	−0.0005 (4)
C1	0.0318 (5)	0.0421 (6)	0.0288 (5)	−0.0053 (5)	0.0044 (4)	0.0008 (4)
C2	0.0345 (6)	0.0538 (7)	0.0320 (6)	−0.0018 (5)	0.0020 (5)	0.0062 (5)
C3	0.0463 (7)	0.0741 (10)	0.0284 (6)	−0.0025 (7)	−0.0008 (5)	0.0013 (6)
C4	0.0527 (8)	0.0683 (9)	0.0339 (7)	−0.0054 (7)	0.0063 (6)	−0.0129 (6)
C5	0.0445 (7)	0.0480 (7)	0.0394 (7)	−0.0032 (6)	0.0087 (5)	−0.0069 (5)
C6	0.0343 (6)	0.0409 (6)	0.0304 (5)	−0.0046 (5)	0.0050 (4)	−0.0002 (5)
C7	0.0351 (6)	0.0394 (6)	0.0332 (6)	−0.0019 (5)	0.0043 (4)	0.0043 (5)
C8	0.0388 (6)	0.0462 (7)	0.0292 (6)	−0.0026 (5)	−0.0007 (5)	0.0020 (5)
C9	0.0350 (6)	0.0451 (6)	0.0315 (6)	−0.0054 (5)	0.0015 (5)	−0.0041 (5)
C10	0.0469 (7)	0.0455 (7)	0.0485 (8)	0.0067 (6)	0.0029 (6)	0.0036 (6)
C11	0.0417 (7)	0.0592 (8)	0.0429 (7)	0.0056 (6)	0.0025 (6)	0.0124 (6)

### Geometric parameters (Å, º)

**Table:**

O1—C9	1.2555 (14)	C4—H4	0.9300
O2—C7	1.3387 (15)	C5—C6	1.4079 (18)
O2—H2	1.09 (2)	C5—H5	0.9300
N1—C9	1.3610 (15)	C6—C7	1.4445 (16)
N1—C1	1.3733 (15)	C7—C8	1.3622 (17)
N1—C10	1.4711 (16)	C8—C9	1.4279 (17)
C1—C6	1.3855 (17)	C8—H8	0.9300
C1—C2	1.3930 (16)	C10—C11	1.547 (2)
C2—C3	1.3721 (19)	C10—H10B	0.9700
C2—C11	1.5104 (19)	C10—H10A	0.9700
C3—C4	1.401 (2)	C11—H11B	0.9700
C3—H3	0.9300	C11—H11A	0.9700
C4—C5	1.3780 (19)		
			
C7—O2—H2	109.2 (11)	O2—C7—C8	123.24 (11)
C9—N1—C1	121.90 (10)	O2—C7—C6	117.37 (11)
C9—N1—C10	126.97 (11)	C8—C7—C6	119.40 (11)
C1—N1—C10	111.12 (10)	C7—C8—C9	123.57 (10)
N1—C1—C6	123.70 (10)	C7—C8—H8	118.2
N1—C1—C2	111.62 (11)	C9—C8—H8	118.2
C6—C1—C2	124.68 (11)	O1—C9—N1	119.54 (11)
C3—C2—C1	117.58 (13)	O1—C9—C8	124.72 (11)
C3—C2—C11	133.96 (12)	N1—C9—C8	115.74 (11)
C1—C2—C11	108.44 (11)	N1—C10—C11	104.24 (10)
C2—C3—C4	119.38 (12)	N1—C10—H10B	110.9
C2—C3—H3	120.3	C11—C10—H10B	110.9
C4—C3—H3	120.3	N1—C10—H10A	110.9
C5—C4—C3	122.35 (13)	C11—C10—H10A	110.9
C5—C4—H4	118.8	H10B—C10—H10A	108.9
C3—C4—H4	118.8	C2—C11—C10	104.54 (10)
C4—C5—C6	119.32 (13)	C2—C11—H11B	110.8
C4—C5—H5	120.3	C10—C11—H11B	110.8
C6—C5—H5	120.3	C2—C11—H11A	110.8
C1—C6—C5	116.67 (11)	C10—C11—H11A	110.8
C1—C6—C7	115.50 (10)	H11B—C11—H11A	108.9
C5—C6—C7	127.82 (12)		
			
C9—N1—C1—C6	2.51 (18)	C1—C6—C7—O2	176.62 (10)
C10—N1—C1—C6	−178.40 (11)	C5—C6—C7—O2	−2.35 (19)
C9—N1—C1—C2	−177.90 (11)	C1—C6—C7—C8	−3.23 (17)
C10—N1—C1—C2	1.20 (15)	C5—C6—C7—C8	177.81 (12)
N1—C1—C2—C3	179.39 (11)	O2—C7—C8—C9	−178.65 (11)
C6—C1—C2—C3	−1.01 (19)	C6—C7—C8—C9	1.18 (19)
N1—C1—C2—C11	−1.98 (14)	C1—N1—C9—O1	175.65 (11)
C6—C1—C2—C11	177.61 (11)	C10—N1—C9—O1	−3.3 (2)
C1—C2—C3—C4	−0.2 (2)	C1—N1—C9—C8	−4.50 (17)
C11—C2—C3—C4	−178.33 (14)	C10—N1—C9—C8	176.56 (11)
C2—C3—C4—C5	1.2 (2)	C7—C8—C9—O1	−177.46 (12)
C3—C4—C5—C6	−1.2 (2)	C7—C8—C9—N1	2.70 (18)
N1—C1—C6—C5	−179.39 (11)	C9—N1—C10—C11	179.12 (11)
C2—C1—C6—C5	1.07 (18)	C1—N1—C10—C11	0.08 (15)
N1—C1—C6—C7	1.52 (17)	C3—C2—C11—C10	−179.78 (15)
C2—C1—C6—C7	−178.02 (11)	C1—C2—C11—C10	1.92 (14)
C4—C5—C6—C1	0.04 (18)	N1—C10—C11—C2	−1.19 (14)
C4—C5—C6—C7	179.00 (12)		

### Hydrogen-bond geometry (Å, º)

**Table:**

*D*—H···*A*	*D*—H	H···*A*	*D*···*A*	*D*—H···*A*
O2—H2···O1^i^	1.09 (2)	1.51 (2)	2.5922 (13)	172.0 (19)

Symmetry code: (i) −*x*, *y*+1/2, −*z*+1/2.

## References

[bb1] Agilent (2011). *CrysAlis CCD* and *CrysAlis RED*, Agilent Technologies, Yarnton, England.

[bb2] Allen, F. H., Kennard, O., Watson, D. G., Brammer, L., Orpen, A. G. & Taylor, R. (1987). *J. Chem. Soc. Perkin Trans. 2*, pp. S1–19.

[bb3] Baumer, V. N., Shishkin, O. V., Ukrainets, I. V., Sidorenko, L. V. & Kayal, S. A. E. (2004). *Acta Cryst.* E**60**, o2356–o2358.

[bb4] Farrugia, L. J. (2012). *J. Appl. Cryst.* **45**, 849–854.

[bb5] Jönsson, S., Andersson, G., Fex, T., Fristedt, T., Hedlund, G., Jansson, K., Abramo, L., Fritzson, I., Pekarski, O., Runstrom, A., Sandin, H., Thuvesson, I. & Björk, A. (2004). *J. Med. Chem.* **47**, 2075–2088. 10.1021/jm031044w 15056005

[bb6] Kutyrev, A. & Kappe, T. (1997). *J. Heterocycl. Chem.* **34**, 969–972.

[bb7] Sheldrick, G. M. (2008). *Acta Cryst.* A**64**, 112–122. 10.1107/S0108767307043930 18156677

[bb8] Ukrainets, I. V., Golik, N. Yu., Andreeva, X. V. & Gorokhova, O. V. (2010). *Chem. Heterocycl. Compd*, **46**, 1459–1466.

[bb9] Ukrainets, I. V., Golik, N. Yu., Shemchuk, A. L., Naboka, O. I., Voronina, Yu. V. & Turov, A. V. (2011). *Chem. Heterocycl. Compd*, **47**, 826–832.

[bb10] Ukrainets, I. V., Sidorenko, L. V., Gorokhova, O. V., Mospanova, E. V. & Shishkin, O. V. (2006). *Chem. Heterocycl. Compd*, **42**, 631–635.

